# Supplementary material for: POViT: Vision Transformer for Multi-Objective Design and Characterization of Photonic Crystal Nanocavities
Source: Nanomaterials (Basel). 2022 Dec 9;12(24):4401. doi: 10.3390/nano12244401 (PMC9784978; doi:10.3390/nano12244401)
Supplement: Supplementary file 1 [file nanomaterials-12-04401-s001.zip › nanomaterials-2013313-supplementary.pdf]

POViT: Vision Transformer for Multi-objective Design and Characterization of Photonic Crystal Nanocavities

Author: Renjie Li, Xinyu Chen, Yueyao Yu. Correspondence should be directed to zhangzy@cuhk.edu.cn

Table S1. Additional Hyper-parameters of POViT

Hyper-parameter	Value
input img size	[B, C, H, W]=[N, 3, 6, 12]
patch size	[B, C, H, W]=[N, 3, 2, 2]
num of patches	$(6 \times 12) \div (2 \times 2) = 18$
patch dim	$3 \times 2 \times 2 = 12$
embedded patch dim	36
Transformer dim	$36 \times 18 = 648$
Transformer depth	6
Transformer num of heads	6
Transformer dim per head	12
Transformer mlp dim	72
pooling method	cls
output size	[B, O]=[N, 2]

Table S2. Additional Hyper-parameters of CvT

Hyper-parameter	Value
input channels	3, 12, 24
input img size	$5 \times 12, 3 \times 6, 2 \times 3$
output channels	12, 24, 36
output img size	$3 \times 6, 2 \times 3, 1 \times 2$
patch size	$3 \times 3, 3 \times 3, 2 \times 2$
patch stride	2, 2, 1
patch padding	1, 1, 0
attention depth	3, 2, 1
num of heads	4, 8, 12
mlp ratio	2, 2, 2
dim of mlp head	$36 \times 1 \times 2 = 72$

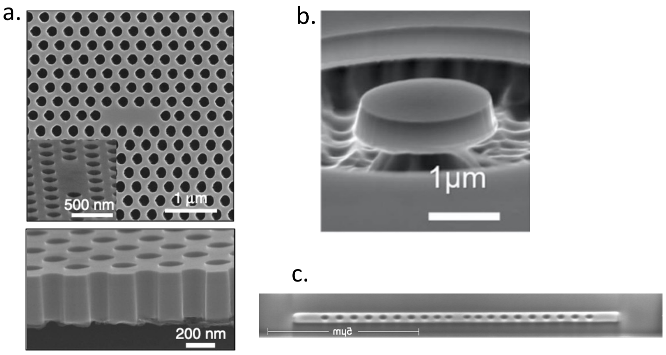

Figure S1. Examples of fabricated nanoscale semiconductor lasers by our group. a: L3 PC nanocavity laser. b: GaAs quantum dot microdisk laser. c: PC nanobeam laser. Photographs taken by Scanning Electron Microscope.

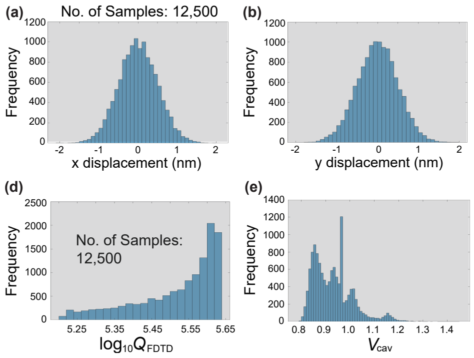

Figure S2. Dataset used for training POViT. Top row: input data. Bottom row: output targets. a. x diplacement, b. y displacement, c. r variation, d. Q factors, and e. mode volumes V.

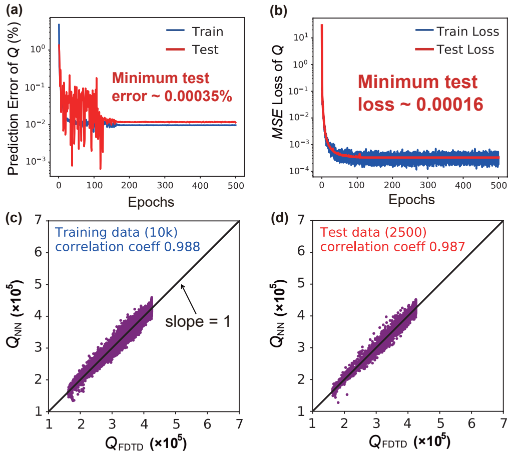

Figure S3. Learning curves and training results of the reproduced (i.e. augmented) CNN model for predicting Q used in this work. a. pred errors, b. MSE loss, c. training correlation coeff of Q, d. test correlation coeff of Q.

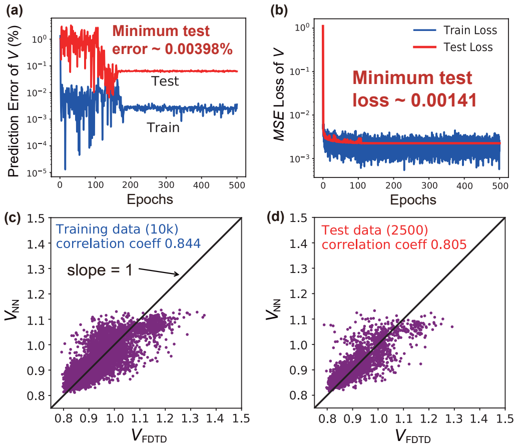

Figure S4. Learning curves and training results of the reproduced (i.e. augmented) CNN model for predicting V used in this work. a. pred errors, b. MSE loss, c. training correlation coeff of V, d. test correlation coeff of V.

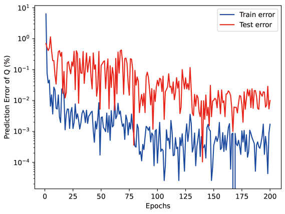

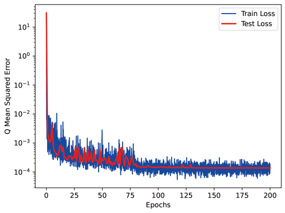

(a) (b)

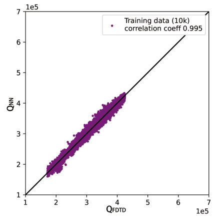

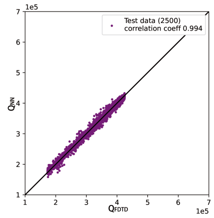

(c) (d)

Figure S5. Learning curves and training results of the CvT model for predicting Q used in this work. a. pred errors, b. MSE loss, c. training correlation coeff of Q, d. test correlation coeff of Q.

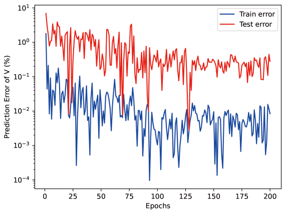

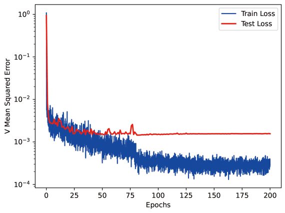

(a) (b)

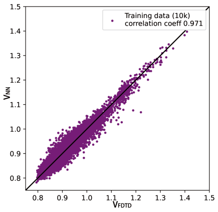

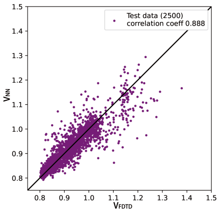

(c) (d)

Figure S6. Learning curves and training results of the CvT model for predicting V used in this work. a. pred errors, b. MSE loss, c. training correlation coeff of V, d. test correlation coeff of V.

Table S3: Excerpt of raw data used for generating Table I in main text.

Lr = 0.00005	ABS1	Qmin	0.00000	Vmin	0.000180
		Qconv	0.00654	Vconv	0.049234
		Qloss	0.000285	Vloss	0.001642
		Q	0.988	V	0.8720
	ABS2	Qmin	0.000147	Vmin	0.01565
		Qconv	0.001328	Vconv	0.24861
		Qloss	0.000282	Vloss	0.002262
		Q	0.9880	V	0.8200
	ABS3	Qmin	0.0000346	Vmin	0.0002702
		Qconv	0.01036	Vconv	0.052529
		Qloss	0.000389	Vloss	0.0001663
		Q	0.9840	V	0.868
	Avg	Qmin	0.0000606	Vmin	0.005367
		Qconv	0.006076	Vconv	0.11167
		Qloss	0.000319	Vloss	0.001856
		Q	0.987	V	0.853
Lr = 0.0001	ABS1	Qmin	0.00000	Vmin	0.001331
		Qconv	0.001549	Vconv	0.08713
		Qloss	0.000198	Vloss	0.001308
		Q	0.992	V	0.889
	ABS2	Qmin	0.00004326	Vmin	0.0002315
		Qconv	0.009837	Vconv	0.02858
		Qloss	0.000192	Vloss	0.001362
		Q	0.992	V	0.891
	ABS3	Qmin	0.0001125	Vmin	0.00001
		Qconv	0.008457	Vconv	0.01229
		Qloss	0.000184	Vloss	0.001246
		Q	0.993	V	0.903
	Avg	Qmin	0.00005193	Vmin	0.0005231
		Qconv	0.006615	Vconv	0.04267
		Qloss	0.000191	Vloss	0.001305
		Q	0.992	V	0.894
Lr = 0.001	ABS1	Qmin	0.00003459	Vmin	0.001471
		Qconv	0.003462	Vconv	0.07320
		Qloss	0.000093	Vloss	0.001260
		Q	0.996	V	0.896
	ABS2	Qmin	0.0000	Vmin	0.00004528
		Qconv	0.006052	Vconv	0.01738
		Qloss	0.000088	Vloss	0.001199
		Q	0.996	V	0.890
	ABS3	Qmin	0.00050191	Vmin	0.0003795
		Qconv	0.006604	Vconv	0.01999
		Qloss	0.000106	Vloss	0.001615
		Q	0.996	V	0.872
	Avg	Qmin	0.0001788	Vmin	0.00063197
		Qconv	0.005373	Vconv	0.03686
		Qloss	0.000096	Vloss	0.001358
		Q	0.996	V	0.886
Lr = 0.01	ABS1	Qmin	0.00005189	Vmin	0.001437
		Qconv	0.003811	Vconv	0.06989
		Qloss	0.000333	Vloss	0.001017
		Q	0.986	V	0.913
	ABS2	Qmin	0.0001125	Vmin	0.0005903
		Qconv	0.005988	Vconv	0.1544
		Qloss	0.000112	Vloss	0.001096
		Q	0.996	V	0.917
	ABS3	Qmin	0.00009	Vmin	0.0006123
		Qconv	0.005850	Vconv	0.03276
		Qloss	0.000201	Vloss	0.001147
		Q	0.992	V	0.907
	Avg	Qmin	0.00005767	Vmin	0.0008799
		Qconv	0.005217	Vconv	0.08568
		Qloss	0.000215	Vloss	0.001087
		Q	0.991	V	0.912

GELU Vcoeff data				Avg
Lr = 0.00005	0.802	0.798	0.796	0.799
Lr = 0.0001	0.819	0.838	0.822	0.826
Lr = 0.0002	0.859	0.869	0.878	0.869
Lr = 0.0005	0.831	0.851	0.855	0.846
Lr = 0.001	0.891	0.869	0.871	0.877
Lr = 0.005	0.889	0.895	0.886	0.890
Lr = 0.01	0.888	0.920	0.917	0.908
Lr = 0.02	0.748	0.763	0.763	0.758
GeLu Qcoeff data				
Lr = 0.01	0.991	0.994	0.994	0.993

GELU Q loss				
0.000227	0.000148	0.000150	→	0.000175
V loss				
0.00139	0.000925	0.001028	→	0.001114
Q min err				
0.0001557	0.0003631	0.0002078	→	0.000242
V min err				
0.000741	0.000887	0.001255	→	0.000961
Q conv err				
0.0.01849	0.01207	0.004292	→	0.01161
V conv err				
0.0.03706	0.03860	0.03333	→	0.03633

continued.

GELU		
0.000122	Min 0.00001731	Coeff 0.995
	Conv 0.0026126	
0.000120	Min 0.00006065	Coeff .995
	Conv 0.0025802	
0.000106	Min 0.00002595	Coeff 0.996
	Conv 0.003666	
Loss	0.000116	
Min	0.000350	
Conv	0.002953	
Coeff	.995	

continued.
